# Biocompatible graphene-zirconia nanocomposite as a cyto-safe immunosensor for the rapid detection of carcinoembryonic antigen

**DOI:** 10.1038/s41598-021-99498-0

**Published:** 2021-11-18

**Authors:** Lih Poh Lin, Shiau-Ying Tham, Hwei-San Loh, Michelle T. T. Tan

**Affiliations:** 1 Department of Electrical and Electronics Engineering, Faculty of Engineering and Technology, Tunku Abdul Rahman University College, Jalan Genting Klang, 53300 Kuala Lumpur, Malaysia; 2 Department of Electrical and Electronic Engineering, Faculty of Science and Engineering, University of Nottingham Malaysia, Jalan Broga, 43500 Semenyih, Selangor, Malaysia; 3 School of Biosciences, Faculty of Science and Engineering, University of Nottingham Malaysia, Jalan Broga, 43500 Semenyih, Selangor, Malaysia

**Keywords:** Biomarkers, Chemistry, Engineering, Nanoscience and technology

## Abstract

Graphene-based materials have gained remarkable attention in numerous disciplines owing to their unique electrochemical properties. Out of various hybridized nanocomposites, graphene-zirconia nanocomposite (GZ) was distinctive due to its biocompatibility. Zirconia nanoparticles serve as spacers that reduce the stacking of graphene and improve the electrochemical performance of the material. Considering that lungs and skin suffer the greatest exposure to nanoparticles, this study aimed to evaluate the cytotoxicity of the as-synthesized GZ nanocomposites on MRC5 (lung cells) and HaCaT (skin cells) via morphological observation and cell viability assay using 3-(4,5 dimethylthiazol-2-yl)-(2,5-diphenyltetrazolium bromide) tetrazolium (MTT). GZ-treated cells showed a comparable proliferation rate and morphology with untreated cells under microscopic evaluation. Based on MTT results, the IC_50_ values of GZ were > 500 µg/ml for MRC5 and HaCaT cells. The excellent biocompatibility was the supremacy of GZ over other nanocomposites applied as electrode materials in biosensors. GZ was functionalized with biolinker for the detection of carcinoembryonic antigen (CEA). The proposed immunosensor exhibited good responses towards CEA detection, with a 4.25 pg/ml LOD and correlation coefficient of R^2^ = 0.99 within a linear working range from 0.01 to 10 ng/ml. The performance of the immunosensor to detect CEA present in human serum was also evaluated. Good recovery of CEA was found, suggesting that the proposed immunosensor possess a high affinity to CEA even in a complex biological matrix, rendering it a promising sensing platform for real sample analysis and open a new way for the detection of cancer-associated proteins.

## Introduction

A disheartening 1.8 million new cancer cases and 600 thousand deaths were estimated for the year 2020 in the US^1^. The actual mortality is expected to be higher due to the barrier to get cancer diagnostic amid a pandemic. The impact of the COVID-19 pandemic has had a dreadful consequence on the delivery of cancer diagnostic and treatment. An alarming finding showing a decrease in cancer diagnostic was reported by Netherlands Comprehensive Cancer Organisation, in which relative diagnosis of cancer was found to be as low as 40% of the usual capacity during the outbreak of COVID-19 in early 2020^2^. Diagnostic evaluations were either postponed or have prolonged waiting times because hospital resources and laboratory facilities were relocated to alleviate the burden on health care caused by COVID-19. As early diagnosis and rapid treatment regimes have been the indispensable factors behind improved cancer survival, the impending mortalities induced by delayed diagnostic is worrying^3^. A feasible solution to address the issue of laboratory shortage is the development of biosensors for rapid cancer diagnosis. Rapid screening offers the opportunity for early detection, which is known to increase the survival rate for cancers of the lung, rectum, colon and breast^4^.

Correspondingly, a glycoprotein involved in cell adhesion, the carcinoembryonic antigen (CEA) is known to be overexpressed particularly in colorectal cancers^5–7^. The CEA level of a healthy individual is ideally below 5.0 ng/ml^8^, therefore serum CEA levels higher than that of healthy persons led to its diagnostic role as a cancer indicator. Although conventional techniques such as the enzyme-linked immunosorbent assay (ELISA) and polymerase chain reaction (PCR) are well documented in CEA detection^9,10^, they still suffer from some drawbacks including the need for trained personnel to perform the test^11^, lengthy assay duration^12^, background noise^13^ and potential false readings associated with the complex coloured sample^14^. These drawbacks are more pronounced during a pandemic where laboratory resources are scarce. In contrast, electrochemical immunoassay, due to its rapidness, specificity and feasibility for miniaturization, has received more attention in recent years^15,16^.

Immunosensor exploits the specific affinity between antibody and antigen to quantify detection, in which electrochemical change induced by the hybridization of antigen–antibody is translated into a measurable signal. Immunosensor has established itself as a promising testing method for CEA, and the recent trend shows that various graphene-based nanocomposites have been incorporated to amplify sensor response and consequently enhance the sensitivity of CEA detection^17–19^. Graphene is a single-layer carbon existing in a two-dimensional hexagonal lattice, and its remarkable qualities such as high surface to volume ratio and outstanding electron transport are valuable for the augmentation of sensors performances^20^. The ease of material treatment and the latency to be synthesized into composite are likewise the driving force behind the popularity of graphene in bio-sensing. Graphene-nanocomposite combines the physiochemical attributes of graphene and its hybridized components to deliver a novel material that has enhanced properties including effective surface area, biocompatibility and electrical conductivity that out-perform graphene and its hybridized component individually^21^. Remarkably, the zirconia nanoparticle is a feasible candidate due to its superior characteristics which include exceptional biocompatibility, stability and uniformity^22,23^. Henceforth, hybridizing the unique and well-defined characteristics of both graphene and zirconia nanoparticles produces a nanocomposite, graphene-zirconia (GZ) that has been recognized to be an enhancement with regards to bio-sensing performance, attributed by the role of zirconia as spacers to reduce the restacking of graphene sheets, as shown in previous works^24,25^.

In conjunction with the broader application of graphene-based nanocomposites, the concern of material-induced cytotoxicity has been raised. Cytotoxicity is the extent to which a material can reduce the viability of cells or in other words, it is the measurement of how harmful a substance is toward living organisms. The two simplest means of material exposure are through the skin (contact) and lung (inhalation). Toxic nanomaterial once inhaled can deposit in the respiratory tract, either directly damage the lung epithelium or diffuse into the bloodstream at the tissue-vessel interface^26^. Meanwhile, dermal contact with toxic material can cause effects ranging from mild redness or eczema to severe conditions including cyanosis, tissue destruction and absorption into the bloodstream^27^. Many nanocomposites are known to be toxic to human cells, yet they are applied in biosensors for enhanced performances^28–30^. The investigation of cytotoxicity was generally absent for nanomaterial-enhanced sensing platforms reported in the literature; and this reflects the negligence of potential hazards from material toxicity, especially during the sensor fabrication process. As lungs, together with skins, suffer the utmost exposure to nanomaterial through inhalation and physical contact, it is imperative to investigate the effects of nanocomposite-cell interaction for these two types of cells^31^.

In this study, a disposable and label-free electrochemical immunosensor modified by GZ nanocomposite for the rapid detection of CEA was fabricated. Our work addresses the negligence of material safety with a cytotoxicity study of the GZ nanocomposite. GZ nanocomposite was incubated with human lung cells (MRC5) and skin cells (HaCaT) to evaluate its biocompatibility. The GZ nanocomposite was then functionalized with biolinker 1-pyrenebutyric-acid-N-hydroxysuccinimide-ester (PYSE), followed by the immobilization of CEA antibody (Ab) to yield an immuno-sensing platform. Charge-transfer resistance (Rct) that increases in response to CEA antigen–antibody hybridization was used as the analytical signal to quantify CEA concentration. Fabrication steps and sensing conditions were optimized to amplify the sensor signal. The analytical performances of the as-fabricated CEA immunosensor, together with its specificity, stability and reproducibility were evaluated. In order to demonstrate the real-sample application, the immunosensor was tested against human serum samples containing various CEA concentrations and the outcome was evaluated. The result of this work provides an insight into the promising potential of a biocompatible GZ-based CEA immunosensor to substitute conventional lab-based assays and provide rapid point-of-care detection of CEA.

## Experiment

### Materials and reagent

The materials and reagent used are stated in supplementary data section S.1.

### Cell culture, microscopic examination of cell morphology and MTT study

For the study of material cytotoxicity, two cell line models, namely the human immortalized keratinocyte (HaCaT), and human lung fibroblast (MRC5) were cultured in a sterile condition. Cell culture procedure was performed in 10 ml of DMEM and RPMI-1640 for HaCaT and MRC5, respectively. The medium was replenished twice a week. In order to facilitate cell growth and survival, fetal bovine serum (10%) and penicillin–streptomycin solution (1%) were included in the T-75 cell culture flasks. Incubation of the cells was performed in a humidified atmosphere in the presence of CO_2_ (5%) at 37 °C. Typically, cells were passaged by trypsinization at 70–80% confluence. The growth medium was aspirated followed by the addition of 0.25% trypsin to promote cells dissociation. Subsequently, the collected cells were re-suspended in 4 ml of growth media, added to 10 ml of complete media and maintained accordingly. The cultured HaCaT and MRC5 cells were introduced to GZ nanocomposite by incubating the cells with re-dispersed GZ of various concentrations (0.5, 2.5, 5, 25, 50, 250, 500 μg/ml). The material-treated cells were seeded into 96-well plates (circa 5,000 cells/well) followed by incubation at 37 °C. At point 24-h, 48-h and 72-h, the 96-well plates were retrieved from the incubator and positioned underneath a microscope for examination of cell morphologies. MTT assay was performed to investigate the cell viability of treated HaCaT and MRC5 cells. MTT was dissolved in phosphate-buffered saline (PBS) to reach a final concentration of 5 mg/ml and was subsequently subjected to filtration sterilization. The cultured cells were plated in 96-well plates and incubated with GZ nanocomposite. At point 24-h, 48-h and 72-h, the cells were washed with PBS (100 µl/well) before being added with MTT solution (10 µl/well) and serum-free media (100 µl/well). The MTT-treated cells were incubated for 4 more hours at 37 °C to induce the reduction of tetrazole to formazan by the living cells. Subsequently, the media and MTT solution were substituted with dimethyl sulfoxide (DMSO) solution to dissolve the reduced formazan into a colored solution which absorbance was measured at 570 nm via VersaMax Elisa Microplate Reader (Molecular Device, USA). The degree of absorbance was relative to the concentration of formazan accumulated on and inside the cell. Since formazan was reduced by living cells, the magnitude of absorbance represented the measurement of cell viability. The cell viability was calculated via Eq. (1), as determined at several time points (24-h, 48-h and 72-h).

1 $$Cell\, viability, \%= \frac{{Absorbance}_{treated sample}-{Absorbance}_{background}}{{Absorbance}_{untreated sample}}\times 100$$

IBM SPSS Statistic was used to perform the one-way ANOVA and comparison test to analyze the statistical significance of data collected from the MTT analysis.

### Fabrication of the immunosensor and electrochemical measurements

**Figure 1:**
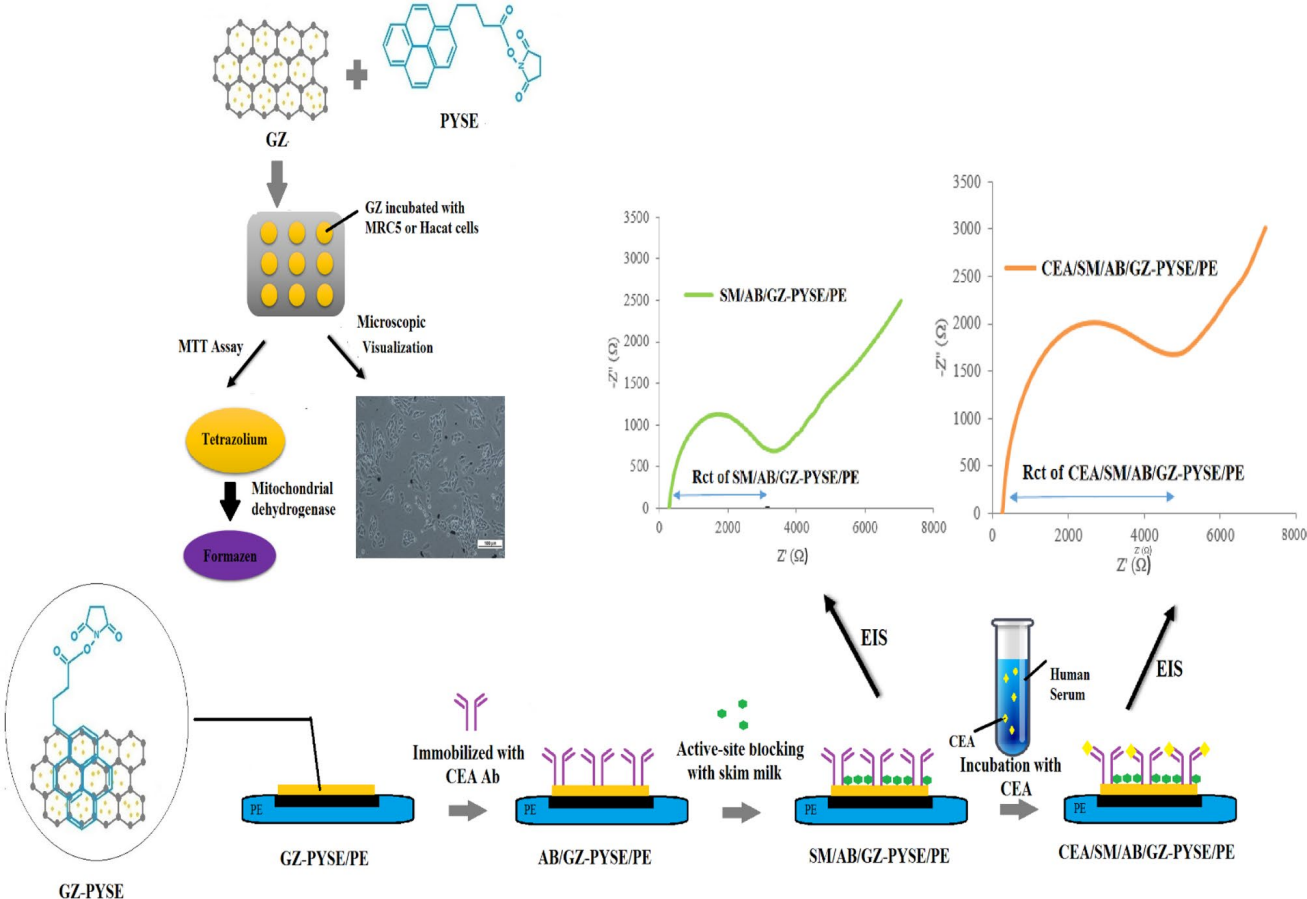
Fabrication of the CEA immunosensor. The detection was governed by the amount of Ab-CEA immuno-complex formed on the electrode surface.

GZ nanocomposite was synthesized according to the protocols established in our previous work^24^ (see supplementary data, Section S.2 for details). The as-synthesized GZ (5 mg) was dispersed in an ethanol–water solvent and mixed with PYSE that would stack on the GZ through π–π interaction. For optimization of the PYSE ratio, GZ: PYSE proportion of 1:2, 1:4, 1:8 and 1:16 was achieved by including 10 mg, 20 mg, 40 mg and 80 mg of PYSE respectively into the mixture. The mixture was sonicated for 30 min in an ultrasonic bath at room temperature. Subsequently, the sediment of the suspension was repeatedly washed to remove unbound PYSE. The PYSE-functionalized GZ was denoted as GZ-PYSE. The GZ-PYSE nanocomposite was then re-dispersed in 5 ml of ethanol–water solvent to yield a suspension consisting of the PYSE-functionalized GZ nanocomposite. Subsequently, 5 µL of the GZ-PYSE suspension was drop-casted on the working electrode of the screen-printed carbon electrode (PE) to yield GZ-PYSE/PE that is ready for antibody immobilization. The GZ-PYSE/PE electrode was washed and left to dry in an ambient condition. Successively, 5 µL of the CEA-Ab (10 µg/ml) was incubated on the working electrode for 50 min before the incubation was halted by rinsing and soaking the electrode in PBS for 5 min. The Ab-immobilized electrode was denoted as Ab/GZ-PYSE/PE. Next, active-site blocking was achieved by incubating the working electrode with 1% skim milk for 20 min, followed by rinsing and soaking of the electrode with PBS to halt incubation. The electrode that has been treated with blocking buffer was denoted as SM/Ab/GZ-PYSE/PE, and it was ready for the detection of CEA. Detection of CEA was achieved by incubating 5 µL of the CEA solution on the working electrode of SM/Ab/GZ-PYSE/PE for 75 min to allow the formation of Ab-CEA immuno-complex. The electrode after Ab-CEA hybridization was denoted as CEA/SM/Ab/GZ-PYSE/PE. The fabrication step of the immunosensor is illustrated in Fig. 1. The electrochemical measurements were completed using Autolab PGSTAT302N potentiostat interfaced with analysis tool NOVA 1.11, in 30 ml of PBS containing the redox species [Fe(CN)6]3^−^/4^−^ (10 mM). Cyclic voltammetry (CV) was logged at 100 mV/s scan rate and swept from—0.4 V until 0.8 V. Nyquist plot was acquired from electrochemical impedance spectroscopy (EIS), performed within the frequency range of 100 kHz to 50 mHz under open circuit potential. EIS considers the diameter of the semi-circle in the high-frequency region crucial wherein it represents the Rct at the sensor-electrolyte interface.

The sensor response is denoted by relative Rct (rRct), which is the relative Rct difference between SM/Ab/GZ-PYSE/PE and CEA/SM/Ab/GZ-PYSE/PE. Equation (2) represents the mathematical expression of rRct.

2 $${\text{rRct }} = \, \frac{{\left( {{\text{Rct}}_{{{\text{CEA}}/{\text{SM}}/{\text{Ab}}/{\text{GZ}} - {\text{PYSE}}/{\text{PE}}}} } \right){-}\left( {{\text{Rct}}_{{{\text{SM}}/{\text{Ab}}/{\text{GZ}} - {\text{PYSE}}/{\text{PE}}}} } \right)}}{{\left( {{\text{Rct}}_{{{\text{SM}}/{\text{Ab}}/{\text{GZ}} - {\text{PYSE}}/{\text{PE}}}} } \right)}}$$

where Rct _SM/Ab/GZ-PYSE/PE_ and Rct _CEA/SM/Ab/GZ-PYSE/PE_ represents the charge transfer resistance of SM/Ab/GZ-PYSE/PE and CEA/SM/Ab/GZ-PYSE/PE respectively.

Every electrochemical measurement was collected in triplicates to provide an averaged value. IBM SPSS Statistic was used to perform the one-way ANOVA and comparison test to analyze the statistical significance of data collected from the optimization steps of sensor fabrication.

## Result and discussion

### Nanomaterial characterization

Raman spectroscopy is a well-recognized technique to evaluate the quality of the as-synthesized graphene. The Raman spectra of graphene, shown in Fig. S1(a) (see supplementary data, section S.3) exhibited three distinctive peaks that are associated with the vibration bands of graphene. These vibrational bands furnished imperative information about graphene quality via the peak position, intensity and sharpness. The band observable at about 1580 cm^−1^ was the G-band (C–C bond stretching) which was attributed to the in-plane vibration of the sp^2^ carbon atoms. Meanwhile, the two peaks at about 1346 cm^−1^ and 2720 cm^−1^ was identified as the D band (‘disorder band’) and the 2D band (‘multipeak feature’) respectively^32^. To evaluate the quality of the as-synthesized graphene, D and 2D peaks were the peak-of-interest as they furnish an insight of graphene quality in terms of exfoliation efficiency or other words, the thickness of the produced graphene. The D peak was usually contributed by the presence of edge defects in graphene, produced during the exfoliation of graphite into graphene. Pronounced D peak reflected the detection of edges and endorsed the efficiency of the exfoliation method. On the other hand, the 2D peak characterized the number of layers of the synthesized graphene. In multi-layer graphene, the 2D peak was usually split into 4 sub-peaks, namely 2D1a, 2D1b, 2D2a and 2D2b^33^. Nonetheless, the distinct peak exhibited by Raman spectroscopy of the graphene synthesized in this work reflected that single-layer graphene was produced. In short, the Raman spectroscopy shows that the ethanol–water exfoliation method employed in this study has efficiently produced thin and pristine graphene sheets. The X-ray diffraction (XRD) result of graphene is shown in Fig S1(b). As evidenced by the dual peaks at 2θ = 26° and 2θ = 55° which corresponded to reflexes (002) and (​004) for graphite (ICSD No. 98-005-2916), graphene with pristine structure was synthesized. These two peaks were also observed in the XRD of graphene-zirconia (GZ) nanocomposite (Fig. S1(c)), endorsing the hybridization of graphene into the nanocomposite. The graphene sheets serve as the backbone for the adhesion of zirconia nanoparticles that could be identified by diffraction at 2θ = 28.06° (plane m(−111)), 2θ = 30.6° (plane t(101)), 2θ = 31.6° (plane m(111)), 2θ = 34.6° (plane m(200)), 2θ = 50.3° (plane t(220), 2θ = 55° (plane m(122)) and 2θ = 60.3° (plane t(311))^25,34^. In short, XRD results suggested that the crystallinity of the nanocomposite was well-preserved. As well, scanning electron microscope (SEM) results showed that synthesis of graphene via exfoliation of graphite was efficacious as supported by the non-opaque form of the graphene sheets shown in Fig. S1(d); and the successful production of GZ nanocomposite can be perceived from the decoration of zirconia nanoparticles on both the surface and edges of graphene, as demonstrated in Fig. S1(e). Energy dispersive spectroscopy (EDS) provided an assessment of the chemical make-up of the GZ composition. Without any impurities, the GZ nanocomposite comprised only of zirconia (peak Z), oxygen (peak O) and carbon (peak C), as shown in Fig. S1(f). The silicon element (peak Si) was caused by the silicon plate used during the scanning. The GZ nanocomposite was also characterized by transmission electron microscopy (TEM). Consistent with observations from SEM, the TEM image showed that the graphene sheet was well exfoliated (Fig. S1(g)), and the graphene sheet was decorated by evenly distributed zirconia nanoparticles (Fig. S1(h)). In a nutshell, all observations manifestly showed that the graphene sheets synthesized were pristine and attachments of unwanted functional groups were absent. At about 10 nM, zirconia nanoparticles were uniformly anchored on the graphene, serving as spaces to reduce restacking of graphene sheets. Non-aggregated graphene would in turn increase the electrochemical performance of the nanocomposite.

### Cytotoxicity effects on lung and skin cells

The investigation of nanomaterial cytotoxicity is generally absent for nanomaterial-enhanced sensing works reported in the literature; this reflects the negligence of the potential hazard triggered by material toxicity since some nanomaterial are recognized to be lethal to human cells^28–30^. As lungs, together with skins, suffer the utmost exposure to nanomaterial through inhalation and physical contact, it is imperative to investigate the effects of nanocomposite-cell interaction for these two types of cells^31^. The cytotoxicity of nanocomposites is essentially influenced by their physical and chemical characteristics determined by the synthesis route and reagent/precursor used^35^. GZ nanocomposite used in this work was synthesized via a green method without reduction steps and harsh chemical doping. It is anticipated that the GZ nanocomposite would possess high biocompatibility owing to its residual-free synthesis method.

#### Microscopic visualization of nanocomposites interaction with HaCaT and MRC5 cells

**Figure 2:**
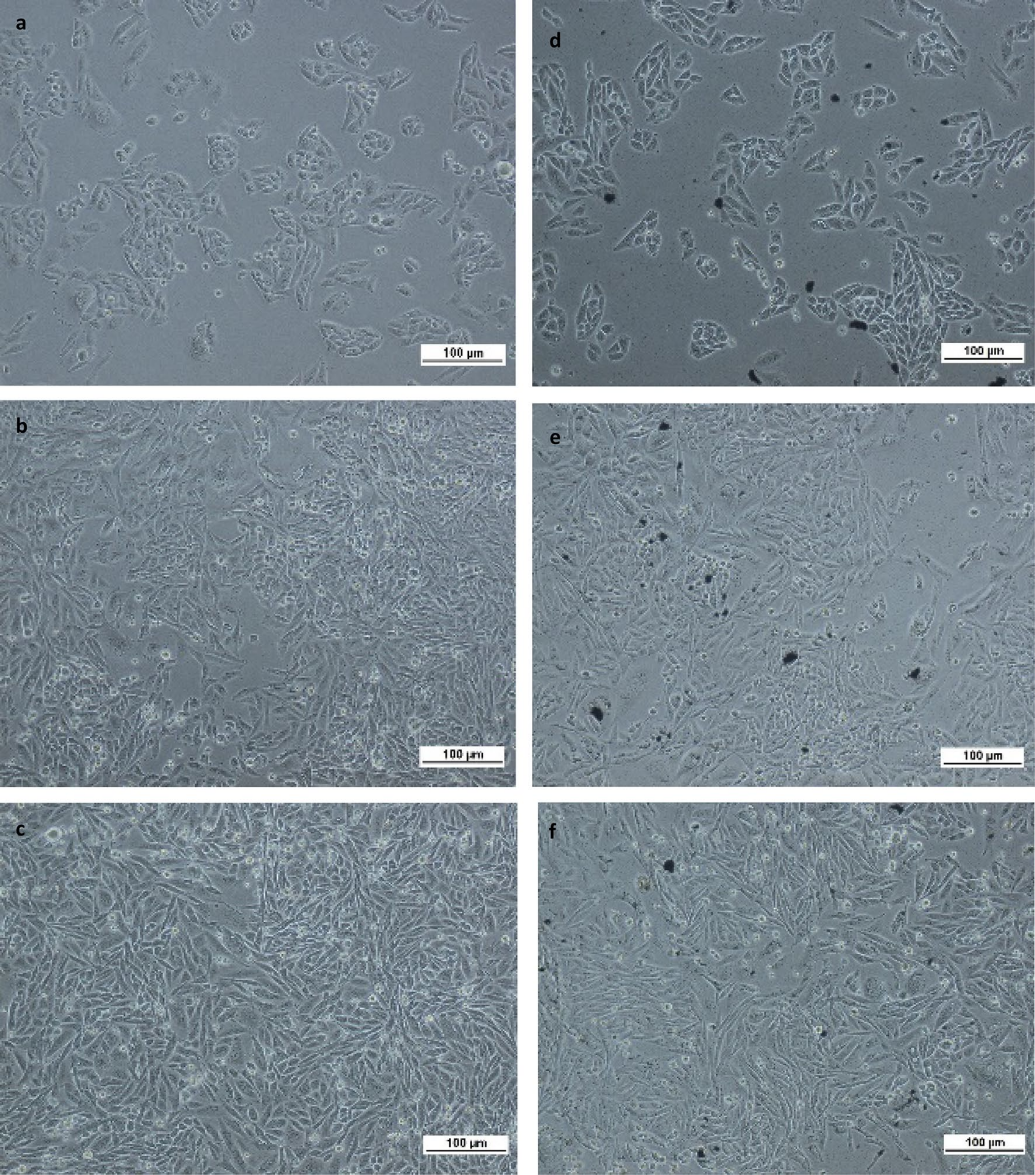
Microscopic characterization of non-GZ-treated MRC5 cells at (**a**) 24 h, (**b**) 48 h, (**c**) 72 h, and GZ-treated MRC5 cells at (**d**) 24 h, (**e**) 48 h, (**f**) 72 h. The presence of GZ nanocomposite did not present an apparent threat to the viability of MRC5 cells. Cells appeared healthy and retained their fibroblastic morphology throughout 72 h of the incubation period.

**Figure 3:**
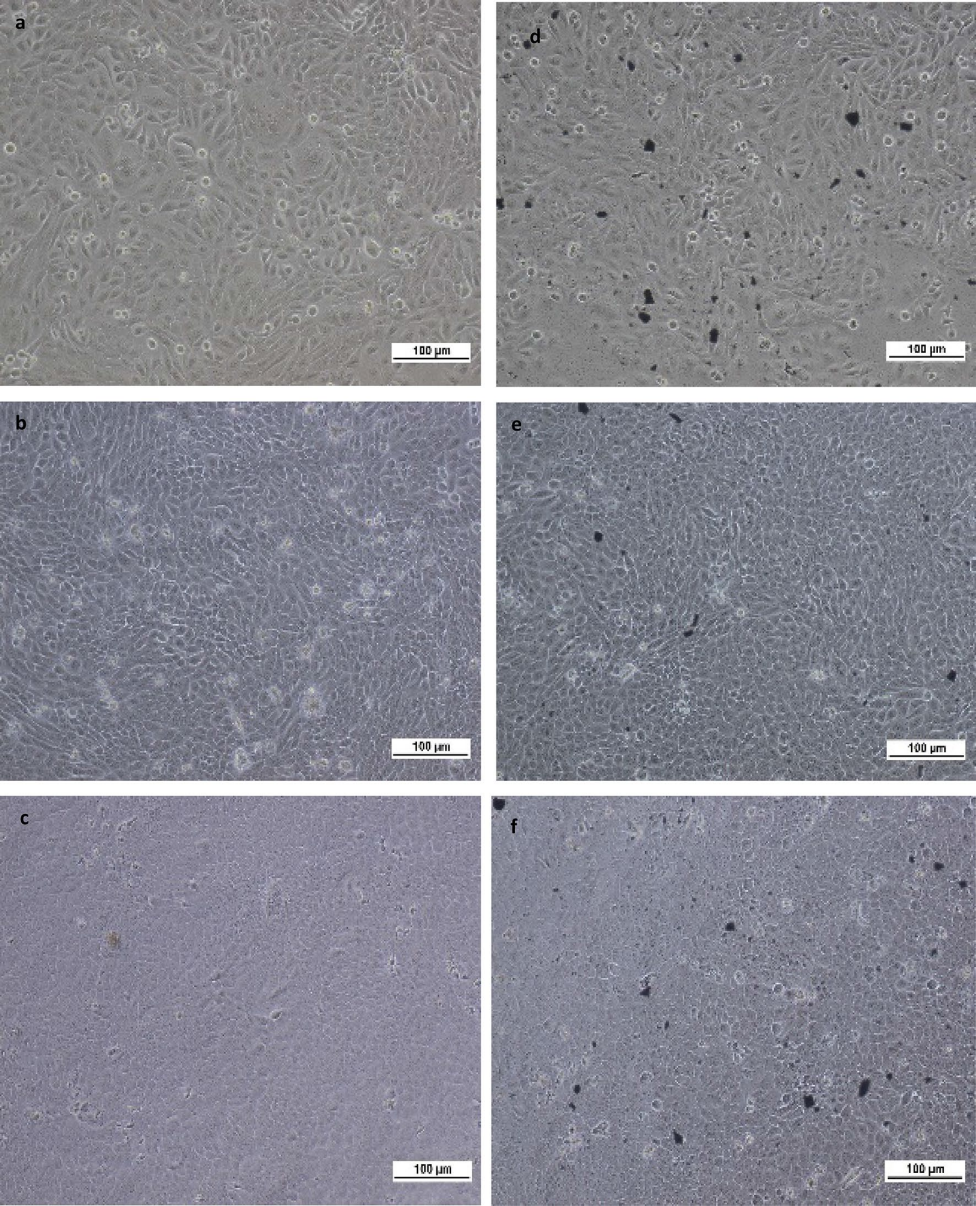
Microscopic characterization of non-GZ-treated HaCaT cells at (**a**) 24 h, (**b**) 48 h, (**c**) 72 h; and GZ-treated HaCaT cells at (**d**) 24 h, (**e**) 48 h, (**f**) 72 h. The presence of GZ nanocomposite did not apparently inhibit the viability of HaCaT cells. Treated cells appeared to be equally proliferative and retained comparable morphology as the control throughout 72 h of the incubation period.

MRC5 and HaCaT are both mammalian cells that grow on substrates. At near 80% confluence, the cells would associate with each other in colonies. MRC5 is a human diploid cell line comprises of fibroblasts derived from human lung tissue. A healthy MRC5 cell has an elongated appearance and a fibroblast-like morphology. Meanwhile, HaCaT, derived from human skin, is an epidermal keratinocyte with a cobblestone appearance. The cell is observed to be rounded and display a high mitotic index. The effect of GZ nanocomposite on MRC5 and HaCaT cell lines are shown in Figs. 2 and 3, respectively. The two characteristics evaluated via the microscope were the apparent cells density (corresponded to proliferation) and the morphology of the cells. Untreated MRC5 cells (Fig. 2a–c) and HaCaT cells (Fig. 3a–c) showed ongoing proliferation and increased density throughout the 72 h of observation. A similar density of cells was observed for MRC5 cells (Fig. 2d–f), and HaCaT cells (Fig. 3d–f) treated with 50 µg/ml of GZ nanocomposite, even after 72 h of incubation. This observation suggested that GZ nanocomposite did not noticeably inhibit the proliferation of both cell lines. The treated cells appeared to be comparably proliferative as the untreated cells. Other than that, typical signs of cell death including granularity around the nucleus, cytoplasmic shrinkage and detachment of the cells from the substrate were not observed for both treated cell lines up to 72 h of incubation, endorsing the low cytotoxicity of the GZ nanocomposite. The morphology of treated MRC5 and HaCaT cells was similar to that of the untreated cells. The presence of GZ nanocomposite did not present a threat to the viability of MRC5 cells in which cells appeared healthy and retained their elongated fibroblastic morphology. Likewise, untreated HaCaT cells and treated HaCaT cells had similar cobblestone morphology throughout the 72 h of observation. In a nutshell, both treated and untreated cell lines exhibited comparable healthy characteristics in terms of proliferation rate and morphology without the sign of necrosis. Therefore, GZ nanocomposite is presumably cyto-safe for these two cell lines.

#### MTT cell viability study

**Figure 4:**
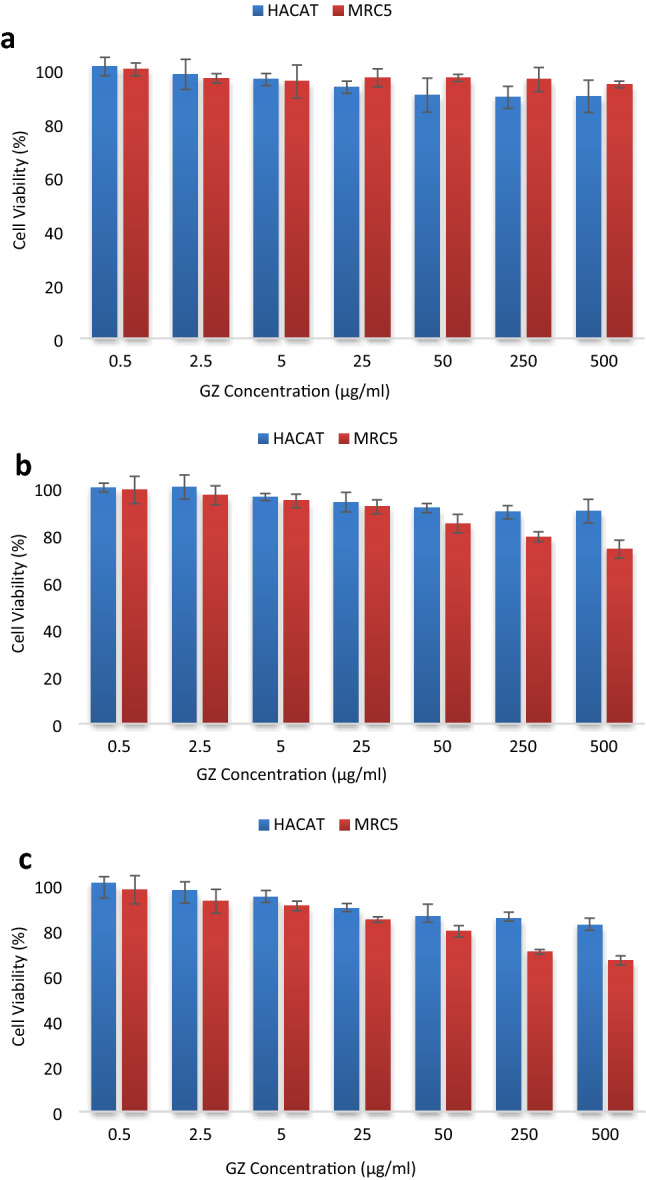
Cell viability of HaCaT and MRC5 cells lines treated with GZ nanocomposite for (**a**) 24 h, (**b**) 48 h, (**c**) 72 h. HaCaT and MRC5 cell lines were generally healthy after incubation with GZ nanocomposite. GZ nanocomposite was considered to be biocompatible for both cell lines.

In parallel to microscopic analysis of the material-cell interaction, cell viability was evaluated via the MTT reduction assay. The MTT assay quantified the mitochondrial metabolic activity of the viable cells, henceforth served as an indication of the intracellular redox state^36^. Only cells retaining normal metabolic function were able to reduce tetrazolium salt to formazan purple solution via the action of mitochondrial dehydrogenase enzyme^37^. Hence, the absorbance of the solution corresponded to the rate of cell survival; a higher rate of cell viability would result in a greater degree of absorbance and vice versa. MTT assessment of the GZ nanocomposite exhibited duration-dependent and dose-dependent toxic effects on cell viability, as shown in Fig. 4. It was observed that a higher concentration of nanocomposite induced greater loss of viability given constant incubation time; meanwhile, at a constant GZ concentration, cell viability declined greater with longer exposure to the material. Based on ISO 10993-5, the percentages of cell viability indicate that > 80% as non-cytotoxic at all; within 60–80% as weakly cytotoxic; within 40–60% as moderate cytotoxic and < 40% as strong cytotoxic to cells^38^.

Notably, the cell viability was observed to be generally high for cells incubated with GZ nanocomposite for only 24 h. As manifested in Fig. 4a, cell viability after 24 h of incubation with 0.5 µg/ml (lowest concentration) and 500 µg/ml (highest concentration) of GZ nanocomposite was observed to be > 99% and > 90% respectively for HaCaT cell line, and > 99% and > 94% respectively for MRC5 cell line, endorsing its non-cytotoxicity according to ISO 10993-5 standard. ANOVA analysis showed that the reduction in cell viability caused by the increase of GZ nanocomposite concentration was statistically insignificant (*p* = 0.834) for MRC5 cells but had met statistical significance (*p* = 0.035) for HaCaT cells. Nonetheless, Dunnett’s comparison test further indicated that the reduction of cell viability of HaCaT cells was significant as compared to the vehicle control (*p* = 0.035) only at GZ concentration ≥ 50 µg/ml. In short, it is established that exposure to a low concentration of GZ nanocomposite (< 50 µg/ml) for a short duration of time (24 h) was practically harmless for both cell lines.

Likewise, as shown in Fig. 4b, cell viability after 48 h of incubation with 0.5–500 µg/ml of GZ nanocomposite was observed to decrease from 99 to 89% for HaCaT cells and 98–74% for MRC5 cells. ANOVA analysis supported the suggestion that the difference in cell viability was statistically insignificant (*p* = 0.224) for HaCaT cells at 48-h time point; Meanwhile, ANOVA and Dunnett’s comparison analysis showed that reduction of cell viability for MRC5 cells was statistically significant (*p* = 0.03) following incubation with GZ concentration of ≥ 50 µg/ml. In summary, at the 48-h time point, it was recognized that HaCaT cells demonstrated resistance to the presence of GZ nanocomposite while MRC5 cells were insignificantly affected for exposure to GZ nanocomposite of less than 50 µg/ml.

As the incubation time was extended to 72 h, as shown in Fig. 4c, the HaCaT cell line was observed to be rather resilient, retaining approximately 82% of cell viability after incubation with 500 µg/ml of GZ nanocomposite, indicating no cytotoxicity. On the other hand, the MRC5 cell line was observed to experience slightly more loss following the same incubation condition, with approximately 67% of cell viability retained, suggesting reasonable biocompatibility (only weak cytotoxicity) on lung cells. ANOVA and Dunnett’s analysis validated that the reduction of cell viability for both cell lines was statistically significant, in which the statistical difference as compared to vehicle control started at a GZ concentration of ≥ 50 µg/ml for HaCat cells (*p* = 0.041) and MRC5 cells (*p* = 0.044).

In summary, the MTT result revealed that the treated HACaT and MRC5 cells were not affected by GZ of < 50 µg/ml up to 72 h of incubation. Supported by high cells viability measured at the 24-h time point, it was also advocated that short-term exposure to GZ was practically harmless to the cells. It was worth noting that a vast majority of the cell populations survived up to 72 h of incubation even with a high concentration of GZ nanocomposite. GZ nanocomposite was deemed safe for biomedical and bio-sensing applications.

The IC_50_ (half maximal inhibitory concentration), is established to be the inhibitory concentration (in this case, the concentration of the GZ nanocomposite) that decreases the cell viability by 50%^39^. It is a comparative index to quantify the toxicity of different materials. A low IC_50_ indicates that a material induces cell death at a low concentration of material exposure and the material concerned is deemed highly toxic to cells; meanwhile, a high IC_50_ suggests that particular material is highly biocompatible in which most cells remain healthy even after exposure to a high concentration of the material. IC_50_ of the GZ nanocomposite was determined to be > 500 µg/ml for both HaCaT and MRC5 cells, or in other words, 500 µg/ml of GZ nanocomposite was well below the toxic level of diminishing half of the cell population. The IC_50_ of GZ was excellent as compared to other nanocomposites. Table 1 summarizes the comparison of IC_50_ for nanocomposites frequently employed in biosensors fabrication. The high cell viability and thus the superior IC_50_ given by the GZ nanocomposite indicated that the nanocomposite was safe on human lung (MRC5) and skin (HaCaT), the two organs most susceptible to nanocomposite toxicity through inhalation and physical contact, respectively. This finding strongly endorsed the application of the as-synthesized GZ for medical and industrial purposes, including the functionalization of the immunosensor demonstrated in this work. While being equally proficient for antigen detection, the immunosensor fabricated in this work was a more superior option considering health and safety requirements. Some other sensors that were fabricated using highly toxic nanomaterials may be hazardous for researchers exposed to the materials through inhalation or physical contact. These materials may also induce severe environmental concerns during mass production. In a nutshell, GZ nanocomposite is a safe and promising electrode material, our proposed immunosensor that employed a cyto-safe material established an upper hand over other reported sensing platforms for the ever-growing field of bio-sensing.

**Table 1:** IC_50_ for nanomaterials commonly used in biosensors.

Nanomaterials	Cell models	Cytotoxicity test	IC_50_	References
Graphene-zirconia	Lung fibroblast, MRC5	24 h MTT assay 48 h MTT assay 72 h MTT assay	IC_50_ > 500 µg/ml IC_50_ > 500 µg/ml IC_50_ > 500 µg/ml	This work
	Human keratinocyte, HaCaT	24 h MTT assay 48 h MTT assay 72 h MTT assay	IC_50_ > 500 µg/ml IC_50_ > 500 µg/ml IC_50_ > 500 µg/ml	
Zirconia	Human monocytic, THP-1	24 h MTT assay	IC_50_ ~ 171.9 µg/ml	^40^
	Rat pheochromocytoma, PC 12	12 h MTT assay 24 h MTT assay 48 h MTT assay	IC_50_ ~ 66.68 µg/ml IC_50_ ~ 53.32 µg/ml IC_50_ ~ 40.82 µg/ml	^41^
	Rat neuroblastoma, N2a	12 h MTT assay 24 h MTT assay 48 h MTT assay	IC_50_ ~ 45.49 µg/ml IC_50_ ~ 33.06 µg/ml IC_50_ ~ 26.27 µg/ml	
Cobalt(II) oxide	Human bronchial epithelial, BEAS-2B	24 h MTT assay	IC_50_ ~ 50 µg/ml	^42^
		24 h Lactate dehydrogenase assay	IC_50_ ~ 100 µg/ml	
Zinc oxide	Human lung adenocarcinoma cells, A549	24 h neutral red uptake assay	IC_50_ ~ 25 µg/ml	^43^
	Human colorectal adenocarcinoma cells, Caco2		IC_50_ ~ 30 µg/ml	
Graphene oxide nanosheet	Human adenocarcinoma alveolar basal epithelial cell line, A549	24 h MTT assay	IC_50_ ~ 100 µg/ml	^44^
Titanium oxide/graphene	Human keratinocyte, HaCaT	24 h MTT assay 48 h MTT assay 72 h MTT assay	IC_50_ = 25 µg/ml IC_50_ = 25 µg/ml IC_50_ = 5 µg/ml	^45^

### Electrochemical characterization of the sensing platform

**Figure 5:**
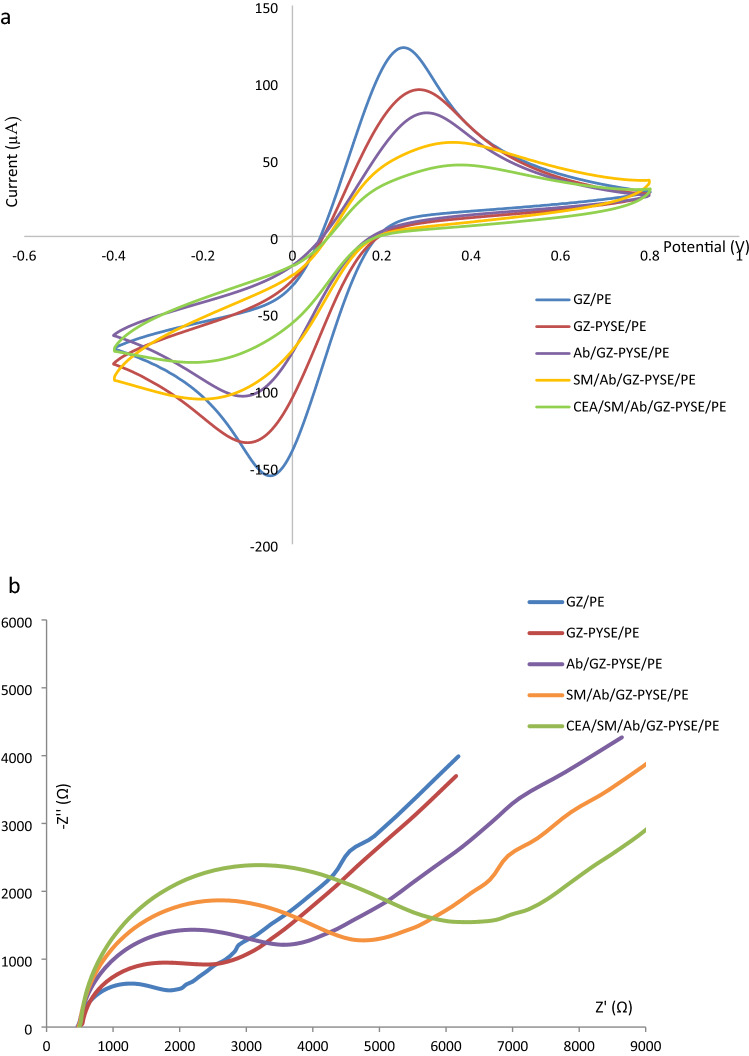
(**a**) CVs and (**b**) EIS of GZ/PE, GZ-PYSE/PE, Ab/GZ-PYSE/PE, SM/Ab/GZ-PYSE/PE and CEA/SM/Ab/GZ-PYSE/PE. Each functionalization step introduced a higher physical barrier at the electrode surface and increased the charge transfer resistance.

CV is an extensively used electrochemical analytical tool that furnishes useful information regarding charge transfer at the sensor interface. CV considers the redox peak current (Ipk) and the inter-peak voltage difference (∆Ipk) as the core characteristic of a sensor. Figure 5a represents the CV of each functionalization step. A pair of distinct redox peaks were observed in the CV assessment of the GZ-functionalized electrode, GZ/PE, occurring at about 0.25 V and − 0.05 V. The Ipk of GZ/PE was the highest among all electrodes, attributed to the large surface area and good conductivity of the GZ nanocomposite. Upon integration of the molecular bi-linker PYSE into the nanocomposite, the Ipk of GZ-PYSE/PE showed a decreased response. It was stipulated that the PYSE, being a hydrophobic element, formed an insulating layer at the sensor-electrolyte interface and contributed to the reduced charge transfer kinetics^46^. Further decrease in the Ipk was observed following the immobilization of GZ-PYSE/PE with the Ab probe (Ab/GZ-PYSE/PE) and the subsequent blocking of active sites with skim milk (SM/Ab/GZ-PYSE/PE). The decrease in Ipk can be ascribed to the agitation of the redox species diffusion due to the obstruction from the insulating layer of protein molecules on the electrode surface^47^. The functionalized sensor SM/Ab/GZ-PYSE/PE was ready for the detection of CEA. The platform was able to hybridize with CEA and instigated a further decrease of Ipk. The decrease advocated the formation of Ab-CEA immuno-complexes that impeded the electron transfer at the sensor-electrolyte interface^48^. Besides, an increasing trend of ∆ Ipk was observed alongside the decreasing pattern of Ipk. The ∆Ipk increased in the order of GZ/PE < GZ-PYSE/PE < Ab/GZ-PYSE/PE < SM/Ab/GZ-PYSE/PE < CEA/SM/Ab/GZ-PYSE/PE. The ∆Ipk represented the degree of redox reversibility, which was the reduction and re-oxidation of redox species. A widening ∆Ipk, where the anodic (cathodic) peak shifted to a more positive (negative) voltage, signified inferior redox reversibility. Every modification step created a more insulative charge transfer barrier so much so that a higher positive (negative) voltage was required to oxidize (reduce) the redox ions and to achieve Nernstian equilibrium^49^. It was obvious that charge-transfer kinetic was more sluggish subsequent to each modification step. In summary, the increasing trend of ∆Ipk together with the decreasing trend of Ipk supported the fact that each functionalization step was successful and the sensing platform was competent to form Ab-CEA immuno-complex for analytical application.

EIS is a sensitive electrochemical assay that can be employed to monitor the biomolecular occurrences at the electrode–electrolyte interface, namely affinity-driven events involving proteins, DNA, nucleic acids or even whole cells. The binding of targets to the biorecognition elements on the electrode lead to conformational change which in turn induce impedance change. EIS assay provides a label-free technique that permits straightforward measurement of biorecognition events, making EIS a widely applied electrochemical method^50,51^. Recent literature presented ultrasensitive biosensors based on EIS that allowed detection down to attomolar (aM) concentrations^52–54^, opening a new avenue to detect proteins and genetic elements that can exist at tremendously low concentrations at the initial stages of a particular disease. Figure 5b shows the impedance spectrum of each functionalization layer. The Rct of the GZ/PE electrode was observed to be about 1.65 kΩ ascribed to the high electron transfer of GZ nanocomposite; nonetheless, after the sequential modification of the nanocomposite with PYSE, Ab immobilization and active-site blocking, the GZ-PYSE/PE, Ab/GZ-PYSE/PE and SM/Ab/GZ-PYSE/PE electrodes exhibited increased Rct of 2.4 kΩ, 3.2 kΩ and 4.7 kΩ respectively. This observation was consistent with that obtained from CV measurement in which each functionalization step deposited more molecules on the electrode surface and augmented the barrier for charge transfer at the sensor interface. The SM/Ab/GZ-PYSE/PE electrode once incubated with the targeted CEA yielded electrode CEA/SM/Ab/GZ-PYSE/PE that demonstrated Rct of 6.1 kΩ, a result consistent with CV to show that specific Ab-CEA immuno-complex was formed on the electrode surface and the bulky immuno-complex impeded the diffusion of the redox species to increase Rct^55^.

Besides, the electron transfer rate constant (ETR) can be calculated based on the observed Rct using the equation shown in Eq. (3)^56–58^.

3 $$\mathrm{ETR}=\frac{RT}{A{R}_{Ct}S{n}^{2}{F}^{2}}$$

where R is the gas constant, T is the absolute temperature, A is the working area of the electrode, S is the redox ion’s concentration, n is the number of electrons involved, and F is the Faraday constant. The ETR, calculated for each electrode according to the functionalization sequence, exhibited a decreasing trend, with CEA/SM/Ab/GZ-PYSE/PE demonstrated the lowest ETR among all the electrodes (3.48 × 10^−5^ cm/s), endorsing that its faradaic process was the slowest among all the electrodes owing to the bulky Ab-CEA immuno-complex on the electrode surface^57^. In brief, ETR decreased in the order of GZ/PE (1.26 × 10^−4^ cm/s) > GZ-PYSE/PE (8.82 × 10^−5^ cm/s) > Ab/GZ-PYSE/PE (6.68 × 10^−5^ cm/s) > SM/Ab/GZ-PYSE/PE (4.53 × 10^−5^ cm/s) > CEA/SM/Ab/GZ-PYSE/PE (3.48 × 10^−5^ cm/s). The consistently decreased ETR was coherent with the continually decreased Ipk in CV since the charge transfer kinetic governed the magnitude of Ipk. In a nutshell, a strong agreement between EIS analysis and CV analysis was observed, and this coherency distinctly signified the effective fabrication of an immunosensor for CEA detection.

To demonstrate that the increment of Rct was specifically caused by the binding of antibody-antigen binding, biolinker-free electrodes (which made the immobilization of CEA-AB infeasible) were tested against 0.1 ng/ml of CEA. It was observed that the Rct difference before and after the CEA exposure was negligible (< 2%), On the other hand, the electrode with immobilized CEA-Ab (SM/Ab/GZ-PYSE/PE) shown approximately 68% of Rct increase when exposed to 0.1 ng/ml of CEA. Intrinsically, the results supported the fact that the increment of Rct was indeed a response to the binding of CEA antibody-antigen.

### Optimization of fabrication parameters and sensing conditions

The sensor response can be amplified through optimization of the fabrication parameters, and sensing conditions, namely the GZ: PYSE proportion, the Ab concentration, the Ab immobilization time, the active-site blocking time, and the Ab-CEA hybridization time. In addition to signal amplification, the optimization steps as well aim to reduce wastage and prevent unproductive states caused by overly dense electrode surfaces that hindered antigen–antibody hybridization. The adopted value for GZ: PYSE, Ab concentration, Ab immobilization time, active-site blocking time and Ab-CEA hybridization time was 1:4, 10 µg/ml, 50 min, 20 min and 75 min, respectively, for enhanced sensor performance. See supplementary data, section S.4 for optimization details.

### Analytical performance, specificity, stability and reproducibility

**Figure 6:**
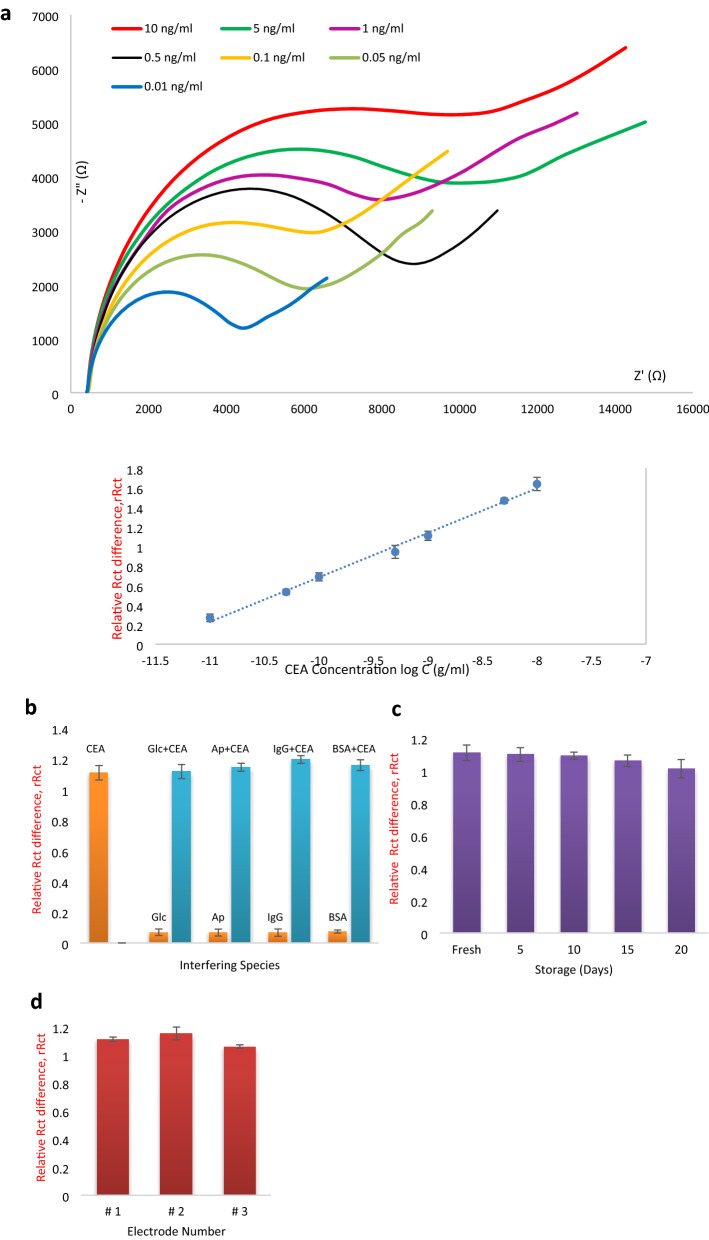
(**a**) EIS of CEA/SM/Ab/GZ-PYSE/PE tested with different concentrations of CEA ranging from 0.01 to 10 ng/ml. Inset: linear correlation between rRct and logarithmic value of CEA concentration, (**b**) specificity study of the immunosensor, (**c**) stability study of the immunosensor and (**d**) reproducibility assessment of the immunosensor. The fabricated immunosensor was specific to CEA and demonstrated reproducibility of 4.27% RSD with good stability. The sensor was able to quantity CEA ranging from 0.01 to 10 ng/ml.

Under optimized sensing conditions, the immunosensor was employed to react with different concentrations of CEA, ranging from 0.01 to 10 ng/ml. Along with the increase of CEA concentration, the impedance at the electrode surface increased due to the greater formation of Ab-CEA immuno-complexes, as shown in the EIS spectra of Fig. 6a. The immuno-complexes serve as the obstructive structures that impeded the diffusion of redox species. The quantification signal, rRct was found to be linearly associated with the logarithmic value of the CEA concentration (Inset of Fig. 6a), with a correlation coefficient of R^2^ = 0.9944 and a calibration equation of rRct = 0.46 log C + 5.24. The limit of detection (LOD) and the limit of quantification (LOQ) of the sensor was estimated to be 4.25 pg/ml (S/N = 3) and 12.89 pg/ml respectively. The concentration of CEA in a healthy individual is within the range of 2.5–5.0 ng/ml wherein a majority of research works took CEA > 5 ng/ml as the cutoff level for possible malignant conditions^59^. The working range of the sensor fitted well into the physiological range of CEA concentration, useful for early detection of CEA over-expression.

Sensor specificity is of utmost importance as it outlines the ability of the developed immunosensor to distinguish CEA from other interfering species specifically. As such, the specificity of the immunosensor was evaluated by measuring the rRct in the presence of other interfering species such as glucose (Glc), acetaminophen (Ap), Immunoglobulin G (IgG) and bovine serum albumin (BSA). The immunosensor was initially tested against samples containing only the interfering species. As shown in Fig. 6b, the rRct measured in interference-only samples were insignificant, demonstrating inertness towards non-CEA species. It was also observed that interfering species that co-exist with CEA did not present substantial influence on the rRct value, where the deviation of rRct as compared to that measured from the CEA-only sample ranged only from 1 to 8%. The inertness towards non-CEA species and the selectivity towards CEA that co-existed with interfering species, evidenced the good specificity of the as-developed immunosensor. Then, the stability of the immunosensor was evaluated. Since this work aims to develop an economical and disposable sensing platform for the rapid testing of CEA, the fabricated immunosensor should be stable under proper storage to provide a reasonable shelf life. For stability study, a series of functionalized PE was stored under 4 °C and tested with CEA (1 ng/ml) at an interval of 5 days, 10 days, 15 days and 20 days as shown in Fig. 6c. The CEA detection of the stored electrodes was comparable to that of freshly prepared electrodes. Even with prolonged storage of 20 days, the immunosensor retained more than 90% of the signal response. The result implied that the bioactivity of Ab was well preserved and the immunosensor has demonstrated decent stability. Reproducibility is a crucial concern with regards to the practical application of immunosensor. In order to illustrate the reproducibility of the immunosensor, a series of electrodes were concurrently fabricated and incubated with CEA (1 ng/ml), followed by the EIS test. The rRct values measured by the different electrodes were in close agreement as shown in Fig. 6d. A relative standard deviation (RSD) of 4.27% was obtained, demonstrating a comparable if not better reproducibility with works reported in the literature^17,55,60^.

An intra-assay variation study was performed to assess the closeness of agreement between triplicated measurements provided by the sensor within the same detection. Meanwhile, an inter-assay variation study was done to investigate the closeness of agreement between measurements provided by the sensor from three individual/independent detections. The degree to which responses of the sensor vary is known as the coefficient of variation (CVs), as presented in Table S.1 (see supplementary data, section S.5). The intra-assay CVs range from 2.36–4.38% for each concentration of CEA tested (0.01–10 ng/ml), yielding an average intra-assay CVs of 3.66%. Meanwhile, the inter-assay CVs range from 2.40 to 7.08% for each concentration of CEA tested, giving an average inter-assay CVs of 4.92%. Commonly, CVs for intra-assays and inter-assays of not more than 10% is considered acceptable^61^. As such, the detection reported in this work demonstrated no intra-assay nor inter-assay variation, rendering the GZ-based immunosensor a reliable and consistent platform.

The performance of the fabricated immunosensor is compared to other CEA sensing platforms listed in Table 2. These sensors were electrochemical assays that exploited the same concept: the interactions between the bio-recognition element and the CEA induce changes such as electron transfer rate, surface conductivity, impedance, and some others. Nonetheless, different nanomaterial and electrochemical techniques were used by these platforms to achieve signal amplification/detection. The electrochemical immunosensor developed in this work was an impedance-based assay that measured CEA concentration via the impedance difference caused by CEA antibody-antigen binding. The label-free fabrication method, based on the biocompatible GZ produced a sensor with performance that was comparable, if not better than other assays. The sensing platform proposed in this work was equally proficient in antigen detection; nonetheless, it was a more superior option considering biocompatibility. Other advantages of the sensor include its label-free detection, straight-forward protein immobilization method and the feasibility for miniaturization due to its disposable design.

**Table 2:** Analytical performance of the fabricated CEA immunosensor against other reported CEA sensors.

Platform	Technique	Working range (ng/ml)	Detection limit (ng/ml)	Refs.
Anti-CEA/nano-gold/carbon nanotubes-chitosan/glass carbon electrode	DPV	0.30–20	0.01	^62^
Streptavidin-functionalized nitrogen-doped graphene/glass carbon electrode	DPV	0.02–12	0.01	^16^
Ferrocene derivatives/gold nanoparticles/gold electrode	SWV	0.05–20	0.01	^13^
Quantum dot coated silica/lead/sandwiched CEA/gold substrate	SWV	0.05–25	0.005	^63^
Poly(3,4ethylenedioxythiophene):poly(4-styrene sulfonate)/conducting Whatman paper strip	EIS	6–20	2.68	^64^
Anti-CEA/gold-silver nanoparticle coated graphene/glass carbon electrode	CV	0.01–120	0.008	^65^
CEA-Ab/GZ-PYSE modified PE	EIS	0.01–10	0.004	This work

### Detection of CEA in human serum

The competence of the immunosensor to detect CEA present in human serum was evaluated to study its feasibility to function within complex biological matrices. Human serum is recognized to be a complex biological medium containing several components such as organic molecules or protein (insulin, bilirubin, glucose, uric acid, urea, fibrinogen, globulins, albumin), lipids (triglycerides, phospholipids, cholesterol) and ions (Mg^2+^, Ca^2+^, K^+^, Na^+^)^66^. Therefore, it was worthy to challenge an immunosensor against detection in human serum. The recovery test was performed by spiking human serum with CEA to prepare samples of various CEA concentrations (0.01 ng/ml, 0.1 ng/ml, 1 ng/ml and 10 ng/ml). EIS was performed on all samples using the as-fabricated immunosensor. Matrix effect provoked by serum components may affect the signal response; therefore, a standard approach to neutralize matrix effect was through dilution. A 100 × dilution factor to achieve complete elimination of matrix effect was employed as the protocol for the analysis of CEA in human serum. The rRct measured from spiked samples and the calibration equation discussed in section *Analytical performance, specificity, stability and reproducibility* were applied to determine the CEA concentration in each sample. As shown in Table 3, good recoveries were found ranging from 103 to 106%, manifesting close agreement with the actual CEA concentration. The finding suggested that the proposed immunosensor possess a high affinity to CEA even in a complex biological matrix, rendering it a promising sensing platform for real sample analysis.

**Table 3:** Recovery test of CEA in human serum.

Sample	Spiked CEA (ng/ml)	Detected CEA (ng/ml)	Recovery (%)
1	0.01	0.01035	103.5
2	0.10	0.10521	105.2
3	1.00	1.00475	100.5
4	10.0	10.6044	106.0

### Advantages of the proposed immunosensor

To the best of the authors’ knowledge, the study of material cytotoxicity was general absent for the majority of nanomaterial-enhanced sensors reported in the literature. The absence of cytotoxicity tests provoked a worry: safety for the researchers and manufacturers, especially during the sensor fabrication process. Although material cytotoxicity has no direct relationship to the performance of an electrochemical sensor, potential hazards from material toxicity should be addressed. The two most accessible routes of material entry were through the skin (contact) and lung (inhalation), therefore our work performed cytotoxicity tests on human lung (MRC5) and skin (HaCat) cells. Thanks to the scalable and green synthesis method, the GZ nanocomposite was free from residual functional groups resulting in cytotoxicity analysis that showed no observable adverse effects on both cell lines. This finding strongly endorsed the application of the as-synthesized GZ for medical and industrial purposes, including the functionalization of the immunosensor demonstrated in this work. Benchmarked with the performances of other reported immunosensors, the sensing platform proposed in this work was equally proficient in antigen detection; nonetheless, it was a more superior option considering biocompatibility.

Equally beneficial is the Ab-immobilization strategy employed in this study. Immobilization of CEA-Ab onto biocompatibility-tested GZ via PYSE has not been reported in the literature. Immobilization proposed here exploited π–π interaction, the dominating supramolecular force to firmly stack PYSE on graphene sheets^67^; PYSE in turn utilized amines-nucleophilic substitution to form amide bonds with amines on the Ab. The GZ-PYSE-Ab linkage in this work was reliable and direct; It was a significantly more robust immobilization technique in comparison to the physical absorption method that bound proteins hydrophobically or electrostatically resulting in uncontrollable orientation and desorption^68^. The GZ-PYSE-Ab immobilization method was also cheaper and simpler to perform than affinity methods such as immobilization using thiols group and streptavidin–biotin that required costly reagents and alteration of the Ab conformation before immobilization^69^.

Another supplementary benefit of the CEA immunosensor lies in its disposable and label-free nature. Keeping in view the aim to develop a point-of-care sensing platform, the CEA sensor proposed in this work was realized with disposable screen-printed carbon electrodes. Vast attention has been directed to the electrode-printing expertise for bio-sensing applications; yet, many reported CEA sensors resorted to glassy carbon electrodes that required taxing preparation protocols involving surface abrasion and chemical cleaning of the electrode. On top of that, the repeated use of glassy carbon electrodes may expose the sensor to possible contaminations and affect the accuracy of the implemented sensing works^17,18,70^. On the contrary, the disposable printed electrode used in our work allowed rapid usage with only a quick rinse before use, and its single-use practice eliminated the risk for cross-contaminations. Sensing platforms developed with screen-printed electrodes possessed a significant lead over the traditional glassy carbon electrodes owing to the possibility of application in portable and miniaturized systems. Besides, the feasibility to perform point-of-care testing and the simplicity of mass production made our disposable screen-printed electrode platform a more commercially promising platform^71^.

A vast number of CEA sensors engaged labels or tags such as secondary protein labels, fluorescent chemical compounds and magnetic nanomaterial to detect the presence and quantify the concentration of CEA^72–74^. In contrast with labelled methods, label-free sensing platforms measured the physical or chemical changes directly and transduced the changes into quantifiable analytical data. Immunosensor proposed in this study, a label-free platform, measured the formation of Ab-CEA immuno-complex and transduced it to the change in Rct, a quantitative parameter. Label-free detection eliminated experimental errors induced by the labelling tags such as sample degradation during the labelling process, inconsistent labelling due to steric hindrance and the failure to identify versatile tags that function reliably^75,76^. The immunosensor proposed was as proficient as the other sensors in CEA detection, yet it was more economical and simpler to fabricate. Without the intervention of label molecules, label-free CEA immunosensor significantly shorten the required time and effort for assay development, thus enhancing its potential for rapid CEA detection.

## Conclusion

This study presented an immunosensor based on a cyto-safe GZ nanocomposite. The cytotoxicity effect of GZ on HaCat and MRC5 cells was determined to be duration- and concentration-dependent. Both GZ-treated and untreated cell lines exhibited comparable healthy characteristics in terms of proliferation rate and morphology without the sign of necrosis. Also, based on MTT results, the IC_50_ values of GZ was found to be > 500 µg/ml for both cell lines. The excellent biocompatibility was the supremacy of GZ over other nanocomposites applied as electrode materials in biosensor development. The GZ-based sensor was applied as an immunosensor for CEA detection. CEA is a glycoprotein involved in cell adhesion and it is known to be overexpressed particularly in colorectal cancer. The CEA level of a healthy individual is ideally below 5.0 ng/ml, therefore serum CEA levels higher than that of healthy persons led to its diagnostic role as a cancer indicator. GZ nanocomposite was functionalized with biolinker PYSE to immobilize CEA antibody probes. CV and EIS analysis validated the accomplishment of each functionalization step and endorsed the specific formation of CEA immuno-complex upon detection. After the optimization of fabrication steps and sensing conditions, the proposed immunosensor exhibited good responses towards CEA detection. Based on the EIS assay that takes about 5 min to complete a measurement, the sensor exhibited a LOD and LOQ of 4.25 pg/ml and 12.89 pg/ml respectively, and a correlation coefficient of R^2^ = 0.99 within a detection range from 0.01 ng/ml to 10 ng/ml. Furthermore, the immunosensor presented high specificity for distinguishing CEA against interfering species in which the rRct from samples that contain interfering species was only 1–8% deviated from that measured from the CEA-only sample. As well, the immunosensor exhibited decent stability for up to 20 days with a minor response deviation of less than 10% and reasonable reproducibility of 4.27% RSD. The competence of the immunosensor to detect CEA present in human serum was likewise evaluated. Good recovery of CEA was found, with a deviation of less than 6% from actual CEA concentration, suggesting that the proposed immunosensor possess a high affinity to CEA even in a complex biological matrix. The key characteristics of the immunosensor offered appealing merits on their own: the stable and straightforward GZ-PYSE-Ab immobilization technique, the biocompatible GZ electrode material, the scalability and handiness of label-free disposable design and the potential for miniaturization; hence, hybridizing all these merits produced a rapid testing platform for CEA detection, opening a new way for timely detection of malignant disorders.

## Supplementary Information

Supplementary Information.
