# Supplementary material for: Biocompatible graphene-zirconia nanocomposite as a cyto-safe immunosensor for the rapid detection of carcinoembryonic antigen
Source: Sci Rep. 2021 Nov 18;11:22536. doi: 10.1038/s41598-021-99498-0 (PMC8602324; doi:10.1038/s41598-021-99498-0)
Supplement: Supplementary file 1 — Supplementary Information. [file 41598_2021_99498_MOESM1_ESM.pdf]

Supplementary Data

Biocompatible Graphene-Zirconia Nanocomposite as a Cyto-Safe Immunosensor for the Rapid Detection of Carcinoembryonic Antigen

Lih Poh Lin, Shiau-Ying Tham, Hwei-San Loh, Michelle T.T. Tan

S.1 Materials and Reagent

Screen-printed carbon electrodes (PE) were acquired from Metrohm (Spain). Graphite flakes were acquired from Bay Carbon (Michigan, USA). Zirconyl Chloride Octahydrate (precursor for zirconia nanoparticle), phosphate-buffered saline (PBS), fetal bovine serum (FBS), bovine serum albumin (BSA), skim milk powder, human serum, acetaminophen, glucose and 1-pyrenebutyric-acid-N-hydroxysuccinimide-ester (PYSE) were acquired from Merck (USA). Ethanol and redox species potassium ferricyanide $[Fe(CN)6]3^{-}/4^{-}$ were purchased from Evergreen Chemical (Malaysia). Carcinoembryonic antigen (CEA) and antibody for carcinoembryonic antigen (Ab) were purchased from Cloud-Clone Corp (USA). Immunoglobulin G (IgG) was purchased from Thermos Scientific (USA). RPMI-1640 growth media and Dulbecco's Modified Eagle Medium, (DMEM), 1% of penicillin-streptomycin solution, 0.25% trypsin and 3-(4,5-dimethylthiazol-2-yl)-(2,5-diphenyltetrazolium bromide) (MTT) were purchased from Nacalai (Japan). Deionized water (Millipore, USA) was used throughout the study.

S.2 Synthesis of Graphene and Graphene-Zirconia Nanocomposite

Graphene was synthesized via the exfoliation of highly oriented pyrolytic graphite (HOPG). Firstly, 50 mg of HOPG was dispersed in a 100 ml solution of ethanol and DI water (2:3). The resultant mixture was sonicated at room temperature in an ultrasonic bath for 180 minutes to procedure a darkish black suspension. The solution was then centrifuged for 30 minutes and the sediment was dried for approximately 12 hours in an oven to yield graphene. Precursor zirconyl chloride octahydrate (ZrOCl2·8H2O) was dissolved in DI water and stirred for even mixing. Subsequently, dried graphene flakes that have been re-dispersed into ethanol/ DI water solution (2:3) were mixed with the precursor solution and sonicated for 120 minutes to produce a uniform dispersion. The mixture was transferred to a 50 ml Teflon/stainless steel autoclave and heated at 180°C in an oven for 18 hours. The solid sediment was isolated from the solution by centrifugation, washed repeatedly with ethanol/DI water and dried for about 12 hours at 70°C in an oven to yield the graphene-zirconia nanocomposite. When ZrOCl2·8H2O was dissolved into DI water, it hydrolysed into Zr(OH)4 and HCl in the solution. HCL volatilize during the high-heat and high-pressure hydrothermal treatment, resulting in the increased concentration of Zr(OH)4 and zirconia nuclei were formed during the long hour hydrothermal process.

S.3 Nanomaterial Characterization

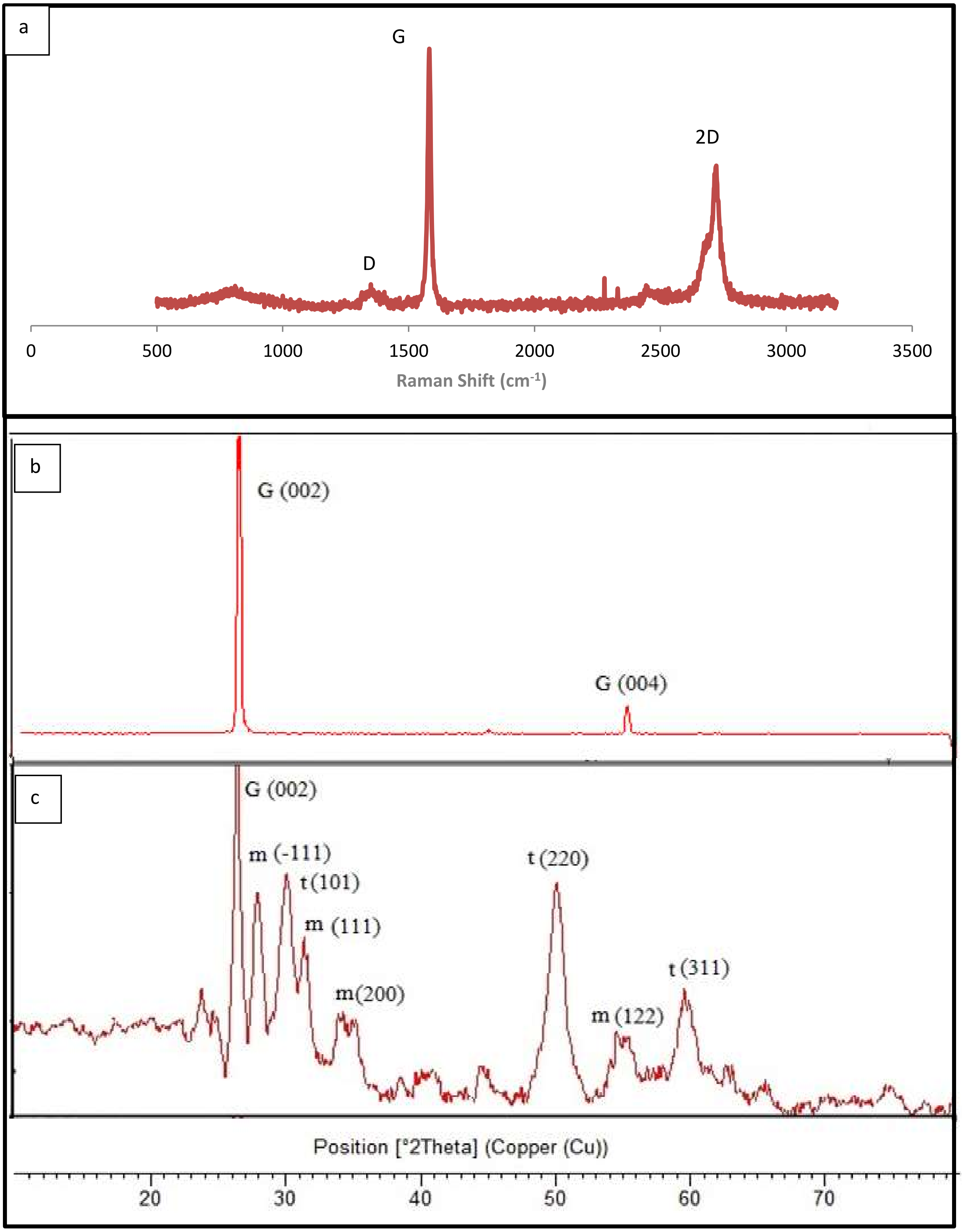

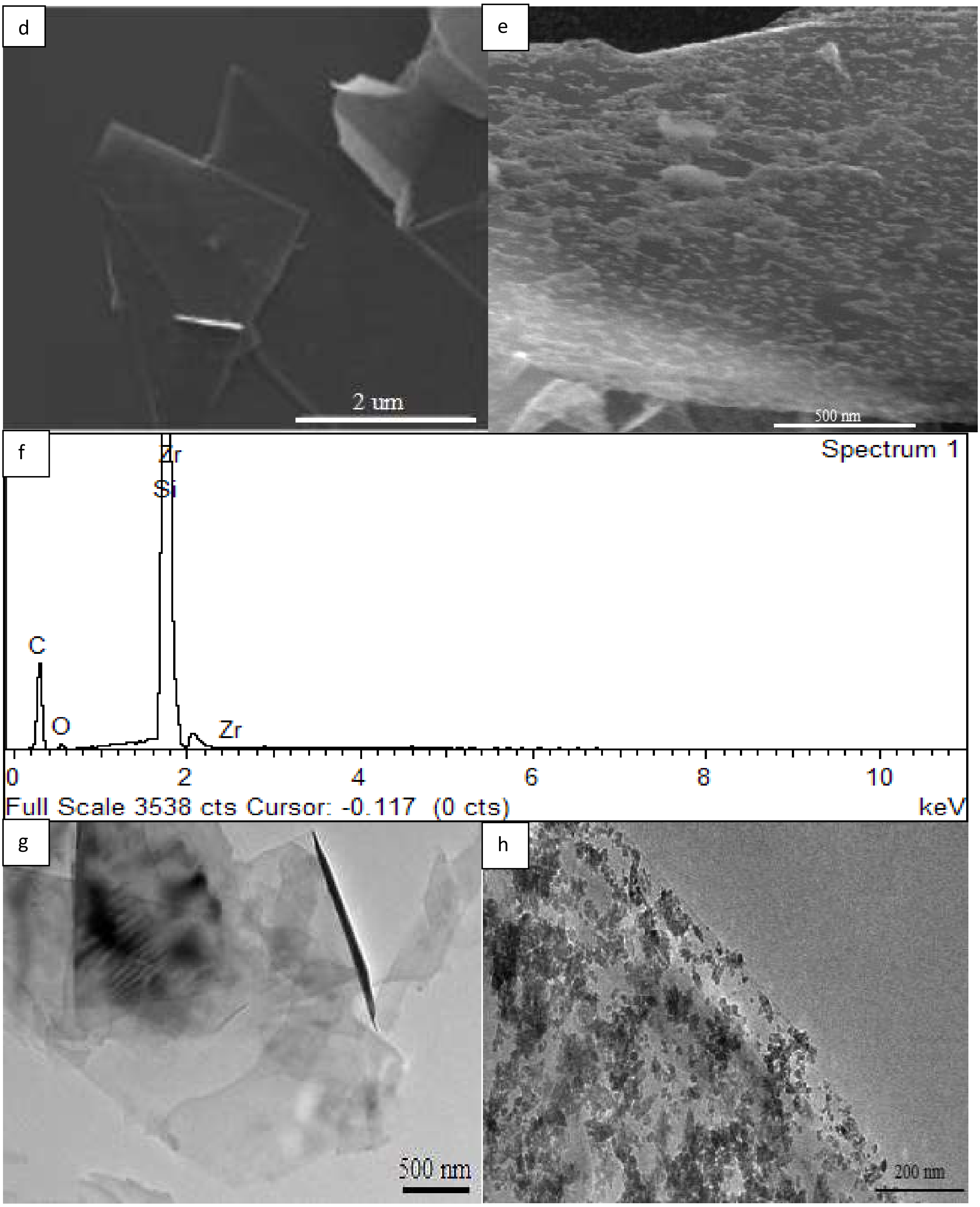

Fig S1 Raman spectra of (a) graphene; XRD of (b) graphene, (c) GZ nanocomposite; SEM images of (d) graphene, (e) GZ nanocomposite; (f) EDS of GZ nanocomposite; and TEM of (g) graphene, (h) GZ nanocomposite

S.4 Optimization of Fabrication Parameters and Sensing Conditions

The sensor response can be amplified through optimization of the fabrication parameters, and sensing conditions viz., the GZ: PYSE proportion, the Ab concentration, the Ab immobilization time, the active-site blocking time, and the Ab-CEA hybridization time. In addition to signal amplification, the optimization steps as well aim to reduce wastage and prevent unproductive states caused by overly dense electrode surfaces that hindered antigen-antibody hybridization.

To determine the optimum GZ: PYSE proportion, electrodes functionalized with different GZ: PYSE nanocomposite (1:2, 1:4, 1:8, and 1:16) were immobilized with 15 μg/ml of Ab for 75 minutes, followed by active-site blocking with 1% skim milk for 20 minutes. The functionalized electrodes were subsequently incubated with 0.5 ng/ml of CEA for 50 minutes. As shown in Fig. S2(a), rRct increased gradually following the adjustment of GZ: PYSE proportion from 1:2 to 1:4, and reduced thereafter. The inferior sensor response at low PYSE proportion was suggestive of inadequate PYSE on the electrode surface. As a result, the amount of immobilized Ab was not efficient for hybridization [5]. On the contrary, high PYSE proportion incorporated abundant amide linkages on the electrode surface and resulted in a thick layer of immobilized Ab. Consequently, the overly dense film of immobilized Ab hindered the hybridization of Ab-CEA due to steric limitations [6]. The finding was validated with statistical analysis using SPSS. ANOVA test showed that GZ: PYSE proportion was a significant parameter affecting the sensor response ($p < 0.05$), while the paired-sample test showed that each adjustment of the PYSE proportion induced a statistically significant change in rRct ($p < 0.05$). Since GZ: PYSE proportion of 1:4 was the most optimum ratio among all electrodes, it was adopted in subsequent analyses.

Likewise, the optimum concentration of Ab was investigated by immobilizing Ab of different concentrations (1 μg/ml, 5 μg/ml, 10 μg/ml, and 15 μg/ml) onto the electrode for 75 minutes, followed by active-site blocking with 1% skim milk for 20 minutes and CEA-incubation for 50 minutes. As shown in Fig. S2(b), rRct increased significantly following higher Ab concentration, reached a maximum at 10 μg/ml and levelled off thereafter. This result is consistent with the finding from the optimization of GZ: PYSE discussed earlier: low concentration of Ab was indicative of overly sparse immobilized Ab for effective CEA detection, whereas high concentration of immobilized Ab induced steric hindrances which would, in turn, limit the Ab-CEA immuno-complex that can be formed [7][8]. Similarly, the ANOVA test endorsed that Ab concentration has a significant influence on rRct ($p < 0.05$), while the paired-sample test confirmed that each adjustment of the Ab concentration was statistically significant on rRct ($p < 0.05$). Since maximum rRct was observed for electrodes immobilized with 10 μg/ml of Ab, this concentration was adopted in subsequent analyses.

Subsequently, the influence of Ab immobilization time was considered for optimum Ab density. Electrodes were immobilized with 10 μg/ml of Ab for varying durations (25 minutes, 50 minutes, 75 minutes and 100 minutes) followed by active-site blocking for 20 minutes and CEA-incubation for 50 minutes. As shown in Fig. S2(c), the rRct for 25 minutes of immobilization time was the lowest among all electrodes. It was stipulated that a certain threshold of time was required for the proteins to form specific binding during incubation [9] and 25 minutes of immobilization time was below-threshold. Afterwards, rRct was observed to increase when the immobilization

time was adjusted to 50 minutes; nonetheless, the subsequent increase to 75 minutes and 100 minutes led to a lower rRct, implying less efficient Ab-CEA hybridization. Although extended incubation time furnished ample interval for Ab to be immobilized well on the sensor, prolonged immobilization time caused steric limitation and competitive binding, which hindered the formation of Ab-CEA immuno-complex [10]. As supported by the ANOVA and paired-sample test, adjustment of the Ab immobilization time was statistically significant on rRct ($p < 0.05$). Since 50 minutes of immobilization generated the most amplified sensor response, it was selected as the optimum immobilization time.

The active-site blocking step was optimized by varying the blocking time. Functionalized electrodes were immobilized with 10 µg/ml of Ab for 50 minutes followed by active-site blocking with 1% skim milk for various blocking times (no blocking, 10 minutes, 20 minutes and 30 minutes). All the electrodes were later subjected to incubation with CEA for 50 minutes. As shown in Fig. S2(d), the electrode without the blocking step presented high rRct due to the presence of non-specific binding [11]. The incubated CEA is bound not only to the Ab but also to the PYSE bi-linker due to the presence of amides on the exterior of CEA, causing inaccurately high sensor response. When the blocking time was increased, rRct reduced and reached a plateau after 20 minutes, manifesting that the protein binding sites were saturated with blocking buffer. This observation was supported by the result from the paired-sample test in which the rRct difference from 20 minutes to 30 minutes was statistically insignificant ($p > 0.05$), indicating that further increase of blocking time imposed no effect on rRct. ANOVA result also supported the fact that optimization of blocking time is statistically meaningful. Hence, 20 minutes of blocking time were selected to suppress the non-specific bindings on the electrode surface.

The Ab-CEA hybridization time was investigated by varying the incubation time with the CEA solution. Electrodes were immobilized with 10 µg/ml of Ab for 50 minutes followed by active-site blocking with 1% skim milk for 20 minutes. The electrodes were incubated with CEA for varying durations (25 minutes, 50 minutes, 75 minutes and 100 minutes). Amplified rRct was observed as the hybridization time was increased from 25 minutes to 75 minutes (Fig. S2(e)), depicting the greater Ab-CEA immuno-complex formation. However, prolonged hybridization time (100 minutes) did not result in further change, which implied that the equilibrium of immuno-reaction and the formation of Ab-CEA immuno-complex was saturated [12]. This observation was validated with the aid of the paired-sample test which showed a statistically significant rRct difference from 25 minutes to 75 minutes ($p < 0.05$) but a statistically insignificant rRct change from 75 minutes to 100 minutes ($p > 0.05$). As such, 75 minutes of hybridization time was considered the plateau and was adopted for subsequent analyses.

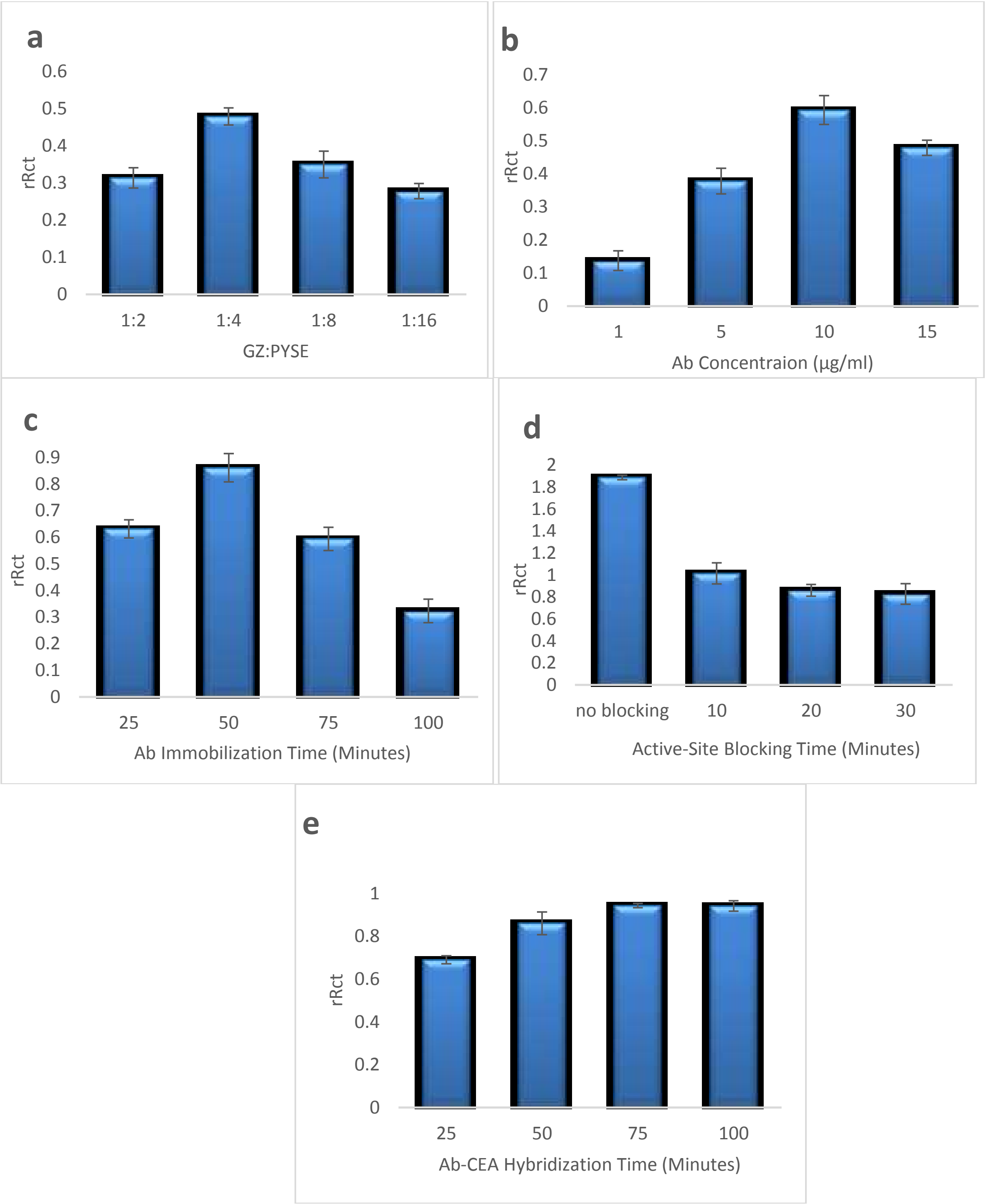

Fig. S2 Influence of (a) GZ: PYSE proportion, (b) Ab concentration, (c) Ab immobilization time, (d) active-site blocking time and (e) Ab-CEA hybridization time on signal response rRct. The adopted value for GZ: PYSE, Ab concentration, Ab immobilization time, active-site blocking time and Ab-CEA hybridization time was 1:4, 10 µg/ml, 50 minutes, 20 minutes and 75 minutes, respectively, for enhanced sensor performance

S.5 Study of Intra-Assay and Inter-Assay Variation

An intra-assay variation study was performed to assess the closeness of agreement between triplicated measurements provided by the sensor within a single detection. Meanwhile, an inter-assay variation study was done to investigate the closeness of agreement between measurements provided by the sensor from three individual/independent detections. The degree to which responses of the sensor vary is known as the coefficient of variation (CVs), as presented in Table S.1.

Table S1: Coefficient of Variation for Intra-Assay and Inter Assay Variation Study

CEA CONCENTRATION (NG/ML)	INTRA-ASSAY CVS (%)	INTER-ASSAY CVS (%)
**0.01**	3.48	6.55
**0.05**	4.38	2.40
**0.10**	3.98	4.84
**0.50**	3.05	7.08
**1.00**	4.30	4.27
**5.00**	4.04	5.19
**10.0**	2.36	4.16
AVERAGE CVS	**3.66**	**4.92**

References

[1] S. Kumar, J.G. Sharma, S. Maji, B.D. Malhotra, Nanostructured zirconia decorated reduced graphene oxide based efficient biosensing platform for non-invasive oral cancer detection, Biosens. Bioelectron. 78 (2016) 497–504. doi:10.1016/j.bios.2015.11.084.

[2] H. Teymourian, A. Salimi, S. Firoozi, A. Korani, S. Soltanian, One-pot hydrothermal synthesis of zirconium dioxide nanoparticles decorated reduced graphene oxide composite as high performance electrochemical sensing and biosensing platform, Electrochim. Acta. 143 (2014) 196–206. doi:10.1016/j.electacta.2014.08.007.

[3] E.R. Ezeigwe, M.T.T. Tan, P.S. Khiew, C.W. Siong, One-step green synthesis of graphene/ZnO nanocomposites for electrochemical capacitors, Ceram. Int. 41 (2014) 715–724. doi:10.1016/j.ceramint.2014.08.128.

[4] A.C. Ferrari, J.C. Meyer, V. Scardaci, C. Casiraghi, M. Lazzeri, F. Mauri, S. Piscanec, D. Jiang, K.S. Novoselov, S. Roth, A.K. Geim, Raman Spectrum of Graphene and Graphene Layers, Phys. Rev. Lett. 97 (2006) 1–4. doi:10.1103/PhysRevLett.97.187401.

[5] P. Khashayar, G. Amoabediny, B. Larijani, H. Morteza, Fabrication and verification of conjugated AuNP-antibody nanoprobe for sensitivity improvement in electrochemical biosensors, Sci. Rep. 7 (2017) 16070. doi:10.1038/s41598-017-12677-w.

[6] A. Makaraviciute, A. Ramanavicius, A. Ramanaviciene, Development of a reusable protein G based SPR immunosensor for direct human growth hormone detection in real samples, Anal. Methods. 7 (2015) 9875–9884. doi:10.1039/C5AY01651G.

[7] K. Omidfar, M. Darzianiazizi, A. Ahmadi, M. Daneshpour, H. Shirazi, A high sensitive electrochemical

nanoimmunosensor based on Fe3O4/TMC/Au nanocomposite and PT-modified electrode for the detection of cancer biomarker epidermal growth factor receptor, Sensors Actuators, B Chem. 220 (2015) 1311–1319. doi:10.1016/j.snb.2015.07.021.

[8] N.G. Welch, J.A. Scoble, B.W. Muir, P.J. Pigram, N.G. Welch, Orientation and characterization of immobilized antibodies for improved immunoassays (Review), Biointerphases. 12 (2017) 02D301-1-02D301-13. doi:10.1116/1.4978435.

[9] J. Han, L. Jiang, F. Li, P. Wang, Q. Liu, Y. Dong, Y. Li, Ultrasensitive non-enzymatic immunosensor for carcino-embryonic antigen based on palladium hybrid vanadium pentoxide / multiwalled carbon nanotubes, Biosens. Bioelectron. 77 (2016) 1104–1111. doi:10.1016/j.bios.2015.11.008.

[10] X. Gu, Z. She, T. Ma, Electrochemical detection of carcinoembryonic antigen, Biosens. Bioelectron. 102 (2018) 610–616. doi:10.1016/j.bios.2017.12.014.

[11] K. Huang, D. Niu, W. Xie, W. Wang, A disposable electrochemical immunosensor for carcinoembryonic antigen based on nano-Au / multi-walled carbon nanotubes – chitosans nanocomposite film modified glassy carbon electrode, Anal. Chim. Acta. 659 (2010) 102–108. doi:10.1016/j.aca.2009.11.023.

[12] Y. Yang, K. Cao, M. Wu, C. Zhao, H. Li, C. Hong, 3D graphene / MWNTs nano-frameworks embedded Ag-Au bimetallic NPs for carcinoembryonic antigen detection, Microchem. J. 148 (2019) 548–554. doi:10.1016/j.microc.2019.05.043.
